# Crystal structure of *N*-(2-hy­droxy-5-methyl­phen­yl)benzamide

**DOI:** 10.1107/S2056989015020575

**Published:** 2015-11-14

**Authors:** Rodolfo Moreno-Fuquen, Nory J. Mariño, Alan R. Kennedy

**Affiliations:** a Departamento de Química – Facultad de Ciencias Naturales y Exactas, Universidad del Valle, Apartado 25360, Santiago de Cali, Colombia; b WestCHEM, Department of Pure and Applied Chemistry, University of Strathclyde, 295 Cathedral Street, Glasgow G1 1XL, Scotland

**Keywords:** crystal structure, benzamide, benzanilide derivatives, biological activity

## Abstract

In the title compound, C_14_H_13_NO_2_, the mean plane of the non-H atoms of the central amide fragment C—N—C(=O)—C (r.m.s. deviation = 0.029 Å) forms dihedral angles of 5.63 (6) and 10.20 (5)° with the phenyl and hy­droxy­phenyl rings, respectively. A short intra­molecular N—H⋯O contact is present. In the crystal, the mol­ecules are linked by O—H⋯O hydrogen bonds to generate *C*(7) chains along [100]. The chains are reinforced by weak C—H⋯O contacts, which together with the O—H⋯O bonds lead to *R*_2_^2^(7) loops. Very weak N—H⋯O inter­actions link the mol­ecules into inversion dimers.

## Related literature   

For the biological activity of benzanilide derivatives, see Calderone *et al.* (2006 ▸). For related structures, see: Gowda *et al.* (2008 ▸); Rodrigues *et al.* (2011 ▸).
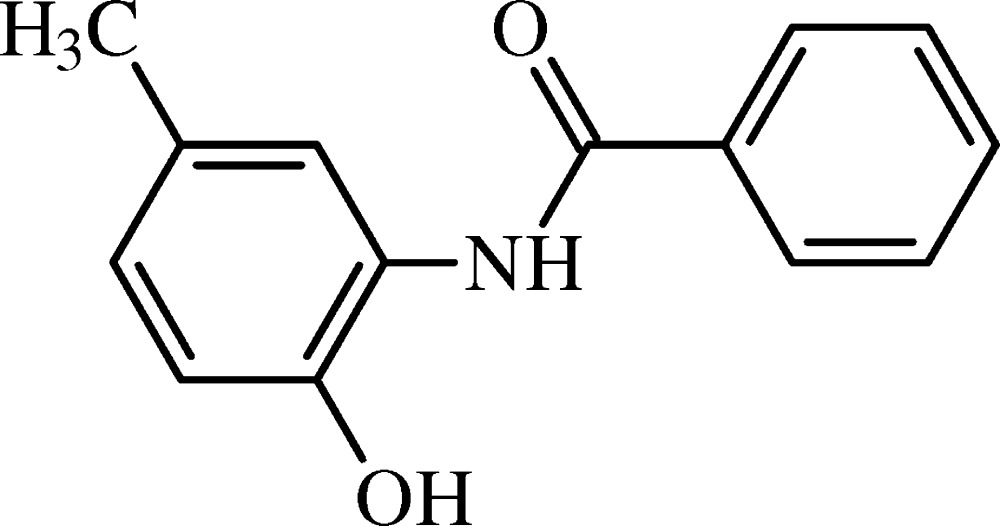


## Experimental   

### Crystal data  

C_14_H_13_NO_2_
*M* *_r_* = 227.25
Monoclinic, 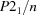

*a* = 7.2263 (3) Å
*b* = 21.7442 (7) Å
*c* = 7.4747 (3) Å
β = 110.280 (5)°
*V* = 1101.69 (8) Å^3^
*Z* = 4
Mo *K*α radiation
μ = 0.09 mm^−1^
*T* = 123 K
0.40 × 0.35 × 0.25 mm

### Data collection  

Oxford Diffraction Gemini S CCD diffractometer
10254 measured reflections
2795 independent reflections
2332 reflections with *I* > 2σ(*I*)
*R*_int_ = 0.032

### Refinement  

*R*[*F* ^2^ > 2σ(*F* ^2^)] = 0.043
*wR*(*F* ^2^) = 0.109
*S* = 1.03
2795 reflections
163 parameters
H atoms treated by a mixture of independent and constrained refinement
Δρ_max_ = 0.33 e Å^−3^
Δρ_min_ = −0.27 e Å^−3^

### 

Data collection: *CrysAlis PRO* (Oxford Diffraction, 2010 ▸); cell refinement: *CrysAlis PRO*; data reduction: *CrysAlis PRO*; program(s) used to solve structure: *SIR92* (Altomare *et al.*, 1994 ▸); program(s) used to refine structure: *SHELXL2014* (Sheldrick, 2015 ▸); molecular graphics: *ORTEP-3 for Windows* (Farrugia, 2012 ▸) and *Mercury* (Macrae *et al.*, 2006 ▸); software used to prepare material for publication: *WinGX* (Farrugia, 2012 ▸).

## Supplementary Material

Crystal structure: contains datablock(s) I. DOI: 10.1107/S2056989015020575/hb7532sup1.cif

Structure factors: contains datablock(s) I. DOI: 10.1107/S2056989015020575/hb7532Isup2.hkl

Supporting information file. DOI: 10.1107/S2056989015020575/hb7532Isup3.cml

**Fig. 1.**
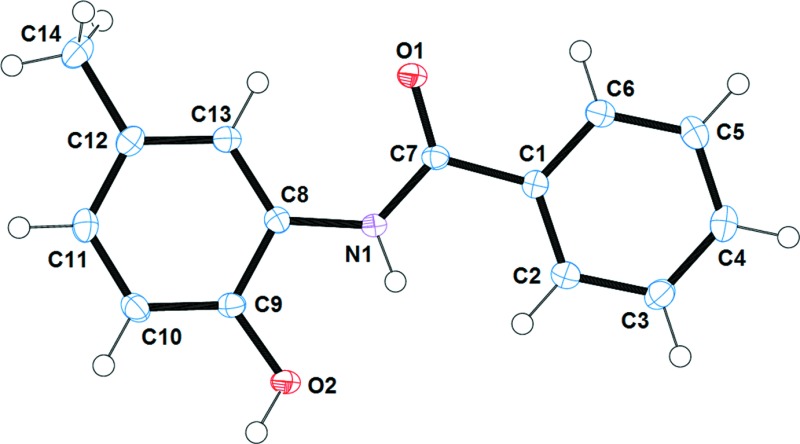
The molecular structure of (I) with displacement ellipsoids drawn at the 50% probability level. H atoms are shown as spheres of arbitrary radius.

**Fig. 2.**
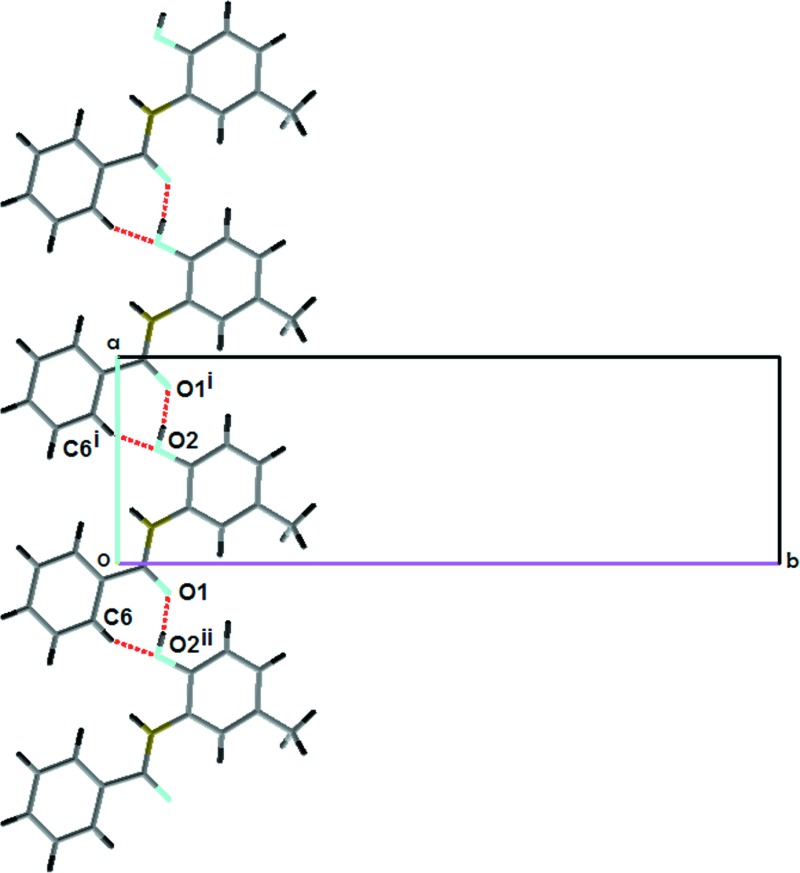
Part of the crystal structure of (I), showing the formation of C(7) chains of molecules along [100] [Symmetry codes: (i) x + 1, +y, +z; (ii) x - 1, +y, +z].

**Fig. 3.**
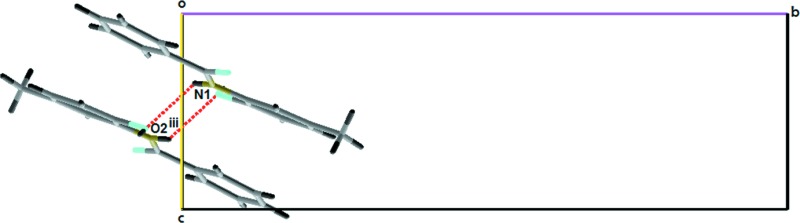
Part of the crystal structure of (I), showing the formation of dimers along [001]. [Symmetry codes: (iii) -x + 1, -y, -z + 1].

. DOI: 10.1107/S2056989015020575/hb7532fig1.tif
The mol­ecular structure of (I) with displacement ellipsoids drawn at the 50% probability level. H atoms are shown as spheres of arbitrary radius.

x y z x y z . DOI: 10.1107/S2056989015020575/hb7532fig2.tif
Part of the crystal structure of (I), showing the formation of C(7) chains of mol­ecules along [100] [Symmetry codes: (i) *x* + 1, +*y*, +*z*; (ii) *x* − 1, +*y*, +*z*].

x y z . DOI: 10.1107/S2056989015020575/hb7532fig3.tif
Part of the crystal structure of (I), showing the formation of dimers along [001]. [Symmetry codes: (iii) −*x* + 1, −*y*, −*z* + 1].

CCDC reference: 1434264

Additional supporting information:  crystallographic information; 3D view; checkCIF report

## Figures and Tables

**Table 1:** Hydrogen-bond geometry (Å, °)

*D*—H⋯*A*	*D*—H	H⋯*A*	*D*⋯*A*	*D*—H⋯*A*
N1—H1⋯O2	0.889 (18)	2.173 (16)	2.6153 (15)	110.0 (13)
N1—H1⋯O2^i^	0.889 (18)	2.518 (17)	3.1928 (14)	133.1 (14)
O2—H20⋯O1^ii^	0.89 (2)	1.75 (2)	2.6390 (12)	171.3 (19)
C6—H6⋯O2^iii^	0.95	2.59	3.4197 (15)	146

Symmetry codes: (i) 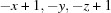; (ii) 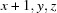; (iii) 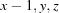.

## supplementary crystallographic information

### S1. Comment

The crystal structure determination of N-(2-hydroxy-5-methylphenyl)benzamide (I), is part of a study on phenylbenzamides carried out in our research group, and it was synthesized from the reaction between of 2-amino-4-methylphenol and benzoyl chloride in acetonitrile. Benzanilide systems have a wide range of biological properties such as potassium channel activators (Calderone *et al.*, 2006). Similar compounds to (I) have been reported in the literature: 2-Methyl-N-(m-tolyl)benzamide (II) (Gowda *et al.*, 2008) and N-(3,5-Dimethylphenyl)-4-methylbenzamide (III) (Rodrigues *et al.*, 2011). The molecular structure of (I) is shown in Fig. 1. The central amide moiety, C8—N1-C7(═O1)—C1, is close to planar (r.m.s. deviation for all non-H atoms = 0.0291 Å) and it forms dihedral angles of 5.63 (6)° with the C1-C6 and 10.20 (5)° with the C8-C13 rings respectively. Bond lengths and bond angles in the molecule are in a good agreement with those found in the related compounds (II) and (III). The conformation of the N—H group is *syn* to the –OH substituent in the benzoyl ring, which results in a short intramolecular N—H···O contact. In the crystal (Fig. 2), molecules are linked by strong O-H···O hydrogen bonds and weak C-H···O intermolecular contacts. Indeed, the O2-H20 at (x,y,z) acts as a hydrogen-bond donor to O1 atom of the carbonyl group at (x+1,+y,+z) and the C6-H6 acts as a hydrogen-bond donor to O2 atom of the hydroxyl group at (x-1,+y,+z). These interactions generate C(7) chains of molecules and *R*_2_^2^(7) rings (See Fig. 2), running along [100]. Additionally, the molecules are linked by N-H···O interactions. N1-H1 acts as a hydrogen-bond donor to O2 atom of the hydroxyl group at (-x+1,-y,-z+1), forming inversion dimers (Fig. 3).

### S2. Experimental

The title molecule was synthesized taking 0.100 g (0.812 mmol) of 2-amino-4-methylphenol dissolved in acetonitrile (10 mL), and then was added benzoyl chloride (0.100 mL, 0.860 mmol). The solution was placed under reflux and constant stirring for 3 hours at 150°C. The solid was filtered and recrystallized from methanol. The solvent was evaporated at room temperature and pink crystals were obtained (m.p. 448 (1)K).

### S3. Refinement

All H-atoms were positioned in geometrically idealized positions, C—H = 0.95 Å, and were refined using a riding-model approximation with *U*_iso_(H) constrained to 1.2 times *U*_eq_ of the respective parent atom. H1 atom was found from the Fourier maps and its coordinates were refined freely.

### Crystal data

**Table:**

C_14_H_13_NO_2_	*D*_x_ = 1.370 Mg m^−^^3^
*M**_r_* = 227.25	Melting point: 448(1) K
Monoclinic, *P*2_1_/*n*	Mo *K*α radiation, λ = 0.71073 Å
*a* = 7.2263 (3) Å	Cell parameters from 10254 reflections
*b* = 21.7442 (7) Å	θ = 3.4–29.4°
*c* = 7.4747 (3) Å	µ = 0.09 mm^−^^1^
β = 110.280 (5)°	*T* = 123 K
*V* = 1101.69 (8) Å^3^	Block, pink
*Z* = 4	0.40 × 0.35 × 0.25 mm
*F*(000) = 480	

### Data collection

**Table:**

Oxford Diffraction Gemini S CCD diffractometer	2332 reflections with *I* > 2σ(*I*)
Radiation source: fine-focus sealed tube	*R*_int_ = 0.032
Graphite monochromator	θ_max_ = 29.4°, θ_min_ = 3.4°
ω scans	*h* = −9→9
10254 measured reflections	*k* = −30→30
2795 independent reflections	*l* = −9→10

### Refinement

**Table:**

Refinement on *F*^2^	Primary atom site location: structure-invariant direct methods
Least-squares matrix: full	Secondary atom site location: difference Fourier map
*R*[*F*^2^ > 2σ(*F*^2^)] = 0.043	Hydrogen site location: mixed
*wR*(*F*^2^) = 0.109	H atoms treated by a mixture of independent and constrained refinement
*S* = 1.03	*w* = 1/[σ^2^(*F*_o_^2^) + (0.0448*P*)^2^ + 0.5141*P*] where *P* = (*F*_o_^2^ + 2*F*_c_^2^)/3
2795 reflections	(Δ/σ)_max_ < 0.001
163 parameters	Δρ_max_ = 0.33 e Å^−^^3^
0 restraints	Δρ_min_ = −0.27 e Å^−^^3^

### Special details

**Table:**

Experimental. The IR spectrum was recorded on a FT—IR SHIMADZU IR-Affinity-1 spectrophotometer. IR (KBr), cm^-1^, 3395 (amide N-H); 3073 (Hydroxyl O-H), 1643 (amide, C=O); 1593 (C=C).
Geometry. All esds (except the esd in the dihedral angle between two l.s. planes) are estimated using the full covariance matrix. The cell esds are taken into account individually in the estimation of esds in distances, angles and torsion angles; correlations between esds in cell parameters are only used when they are defined by crystal symmetry. An approximate (isotropic) treatment of cell esds is used for estimating esds involving l.s. planes.
Refinement. Refinement of *F*^2^ against ALL reflections. The weighted *R*-factor *wR* and goodness of fit *S* are based on *F*^2^, conventional *R*-factors *R* are based on *F*, with *F* set to zero for negative *F*^2^. The threshold expression of *F*^2^ > σ(*F*^2^) is used only for calculating *R*-factors(gt) *etc*. and is not relevant to the choice of reflections for refinement. *R*-factors based on *F*^2^ are statistically about twice as large as those based on *F*, and *R*- factors based on ALL data will be even larger.

### Fractional atomic coordinates and isotropic or equivalent isotropic displacement parameters (Å^2^)

**Table:**

	*x*	*y*	*z*	*U*_iso_*/*U*_eq_	
O2	0.55117 (13)	0.05937 (4)	0.41236 (14)	0.0204 (2)	
O1	−0.14306 (13)	0.07692 (4)	0.30232 (14)	0.0214 (2)	
N1	0.17579 (15)	0.05132 (5)	0.36397 (15)	0.0166 (2)	
C1	−0.07846 (17)	−0.02289 (5)	0.20522 (16)	0.0153 (2)	
C2	0.05257 (19)	−0.06486 (6)	0.17363 (18)	0.0192 (3)	
H2	0.1892	−0.0554	0.2136	0.023*	
C3	−0.0159 (2)	−0.12053 (6)	0.08386 (19)	0.0222 (3)	
H3	0.0743	−0.1490	0.0632	0.027*	
C4	−0.2145 (2)	−0.13469 (6)	0.02438 (18)	0.0217 (3)	
H4	−0.2607	−0.1728	−0.0371	0.026*	
C5	−0.34605 (19)	−0.09320 (6)	0.05467 (19)	0.0224 (3)	
H5	−0.4827	−0.1028	0.0136	0.027*	
C6	−0.27845 (18)	−0.03785 (6)	0.14471 (18)	0.0193 (3)	
H6	−0.3693	−0.0097	0.1656	0.023*	
C7	−0.01844 (17)	0.03897 (5)	0.29485 (17)	0.0150 (2)	
C8	0.26922 (17)	0.10733 (5)	0.43855 (17)	0.0149 (2)	
C13	0.17867 (18)	0.15716 (6)	0.48997 (17)	0.0172 (3)	
H13	0.0430	0.1547	0.4756	0.021*	
C12	0.28408 (19)	0.21075 (6)	0.56243 (18)	0.0186 (3)	
C11	0.48182 (19)	0.21370 (6)	0.58174 (18)	0.0202 (3)	
H11	0.5548	0.2501	0.6301	0.024*	
C10	0.57479 (18)	0.16395 (6)	0.53108 (18)	0.0195 (3)	
H10	0.7104	0.1666	0.5452	0.023*	
C9	0.46985 (17)	0.11075 (5)	0.46028 (17)	0.0157 (2)	
C14	0.1817 (2)	0.26392 (6)	0.6182 (2)	0.0258 (3)	
H141	0.2788	0.2956	0.6812	0.039*	
H142	0.1168	0.2495	0.7059	0.039*	
H143	0.0827	0.2813	0.5038	0.039*	
H1	0.258 (2)	0.0217 (8)	0.357 (2)	0.031 (4)*	
H20	0.658 (3)	0.0685 (9)	0.383 (3)	0.047 (6)*	

### Atomic displacement parameters (Å^2^)

**Table:**

	*U*^11^	*U*^22^	*U*^33^	*U*^12^	*U*^13^	*U*^23^
O2	0.0158 (4)	0.0173 (4)	0.0316 (5)	0.0009 (3)	0.0126 (4)	−0.0013 (4)
O1	0.0150 (4)	0.0177 (4)	0.0333 (5)	0.0005 (3)	0.0108 (4)	−0.0022 (4)
N1	0.0129 (5)	0.0147 (5)	0.0224 (5)	0.0010 (4)	0.0063 (4)	−0.0027 (4)
C1	0.0170 (6)	0.0148 (5)	0.0142 (5)	−0.0004 (4)	0.0056 (4)	0.0008 (4)
C2	0.0161 (6)	0.0200 (6)	0.0210 (6)	0.0009 (5)	0.0056 (5)	−0.0008 (5)
C3	0.0235 (7)	0.0188 (6)	0.0240 (6)	0.0027 (5)	0.0078 (5)	−0.0030 (5)
C4	0.0267 (7)	0.0172 (6)	0.0190 (6)	−0.0028 (5)	0.0051 (5)	−0.0012 (5)
C5	0.0186 (6)	0.0230 (6)	0.0238 (6)	−0.0047 (5)	0.0051 (5)	−0.0013 (5)
C6	0.0162 (6)	0.0197 (6)	0.0220 (6)	0.0008 (5)	0.0065 (5)	−0.0002 (5)
C7	0.0143 (5)	0.0156 (6)	0.0164 (5)	0.0010 (4)	0.0069 (4)	0.0014 (4)
C8	0.0145 (6)	0.0150 (5)	0.0146 (5)	−0.0003 (4)	0.0043 (4)	0.0009 (4)
C13	0.0161 (6)	0.0180 (6)	0.0180 (6)	0.0012 (4)	0.0065 (5)	−0.0001 (4)
C12	0.0227 (6)	0.0155 (6)	0.0178 (6)	0.0015 (5)	0.0073 (5)	0.0006 (4)
C11	0.0230 (7)	0.0149 (6)	0.0222 (6)	−0.0034 (5)	0.0071 (5)	0.0006 (5)
C10	0.0152 (6)	0.0195 (6)	0.0238 (6)	−0.0019 (5)	0.0069 (5)	0.0027 (5)
C9	0.0153 (6)	0.0156 (5)	0.0172 (6)	0.0022 (4)	0.0070 (5)	0.0022 (4)
C14	0.0297 (7)	0.0184 (6)	0.0308 (7)	0.0023 (5)	0.0125 (6)	−0.0044 (5)

### Geometric parameters (Å, º)

**Table:**

O2—C9	1.3667 (14)	C5—H5	0.9500
O2—H20	0.89 (2)	C6—H6	0.9500
O1—C7	1.2369 (14)	C8—C13	1.3871 (16)
N1—C7	1.3440 (15)	C8—C9	1.4038 (16)
N1—C8	1.4103 (15)	C13—C12	1.3949 (17)
N1—H1	0.889 (18)	C13—H13	0.9500
C1—C2	1.3929 (17)	C12—C11	1.3870 (18)
C1—C6	1.3949 (17)	C12—C14	1.5074 (17)
C1—C7	1.4984 (16)	C11—C10	1.3933 (18)
C2—C3	1.3893 (17)	C11—H11	0.9500
C2—H2	0.9500	C10—C9	1.3838 (17)
C3—C4	1.3818 (19)	C10—H10	0.9500
C3—H3	0.9500	C14—H141	0.9800
C4—C5	1.3849 (19)	C14—H142	0.9800
C4—H4	0.9500	C14—H143	0.9800
C5—C6	1.3829 (18)		
			
C9—O2—H20	111.6 (12)	C13—C8—C9	119.61 (11)
C7—N1—C8	128.14 (10)	C13—C8—N1	125.23 (11)
C7—N1—H1	117.3 (11)	C9—C8—N1	115.16 (10)
C8—N1—H1	114.4 (11)	C8—C13—C12	120.90 (11)
C2—C1—C6	118.75 (11)	C8—C13—H13	119.5
C2—C1—C7	123.75 (11)	C12—C13—H13	119.5
C6—C1—C7	117.46 (10)	C11—C12—C13	118.90 (11)
C3—C2—C1	120.22 (12)	C11—C12—C14	121.51 (12)
C3—C2—H2	119.9	C13—C12—C14	119.59 (11)
C1—C2—H2	119.9	C12—C11—C10	120.82 (11)
C4—C3—C2	120.39 (12)	C12—C11—H11	119.6
C4—C3—H3	119.8	C10—C11—H11	119.6
C2—C3—H3	119.8	C9—C10—C11	120.07 (11)
C3—C4—C5	119.83 (12)	C9—C10—H10	120.0
C3—C4—H4	120.1	C11—C10—H10	120.0
C5—C4—H4	120.1	O2—C9—C10	123.73 (11)
C6—C5—C4	119.99 (12)	O2—C9—C8	116.55 (10)
C6—C5—H5	120.0	C10—C9—C8	119.71 (11)
C4—C5—H5	120.0	C12—C14—H141	109.5
C5—C6—C1	120.81 (12)	C12—C14—H142	109.5
C5—C6—H6	119.6	H141—C14—H142	109.5
C1—C6—H6	119.6	C12—C14—H143	109.5
O1—C7—N1	121.94 (11)	H141—C14—H143	109.5
O1—C7—C1	121.11 (11)	H142—C14—H143	109.5
N1—C7—C1	116.95 (10)		
			
C6—C1—C2—C3	−0.16 (18)	C7—N1—C8—C9	−166.27 (11)
C7—C1—C2—C3	−177.86 (11)	C9—C8—C13—C12	0.22 (18)
C1—C2—C3—C4	0.27 (19)	N1—C8—C13—C12	−179.91 (11)
C2—C3—C4—C5	−0.10 (19)	C8—C13—C12—C11	0.28 (18)
C3—C4—C5—C6	−0.2 (2)	C8—C13—C12—C14	−179.59 (12)
C4—C5—C6—C1	0.3 (2)	C13—C12—C11—C10	−0.44 (19)
C2—C1—C6—C5	−0.12 (18)	C14—C12—C11—C10	179.44 (12)
C7—C1—C6—C5	177.72 (11)	C12—C11—C10—C9	0.08 (19)
C8—N1—C7—O1	−5.48 (19)	C11—C10—C9—O2	−178.11 (11)
C8—N1—C7—C1	173.71 (10)	C11—C10—C9—C8	0.44 (18)
C2—C1—C7—O1	172.66 (12)	C13—C8—C9—O2	178.06 (11)
C6—C1—C7—O1	−5.06 (17)	N1—C8—C9—O2	−1.82 (15)
C2—C1—C7—N1	−6.54 (17)	C13—C8—C9—C10	−0.59 (17)
C6—C1—C7—N1	175.74 (11)	N1—C8—C9—C10	179.54 (11)
C7—N1—C8—C13	13.9 (2)		

### Hydrogen-bond geometry (Å, º)

**Table:**

*D*—H···*A*	*D*—H	H···*A*	*D*···*A*	*D*—H···*A*
N1—H1···O2	0.889 (18)	2.173 (16)	2.6153 (15)	110.0 (13)
N1—H1···O2^i^	0.889 (18)	2.518 (17)	3.1928 (14)	133.1 (14)
O2—H20···O1^ii^	0.89 (2)	1.75 (2)	2.6390 (12)	171.3 (19)
C6—H6···O2^iii^	0.95	2.59	3.4197 (15)	146

Symmetry codes: (i) −*x*+1, −*y*, −*z*+1; (ii) *x*+1, *y*, *z*; (iii) *x*−1, *y*, *z*.
