# Supplementary figures and images for: Crystal structure of N-(2-hy­droxy-5-methyl­phen­yl)benzamide
Source: Acta Crystallogr E Crystallogr Commun. 2015 Nov 14;71(Pt 12):o943. doi: 10.1107/S2056989015020575 (PMC4719913; doi:10.1107/S2056989015020575)

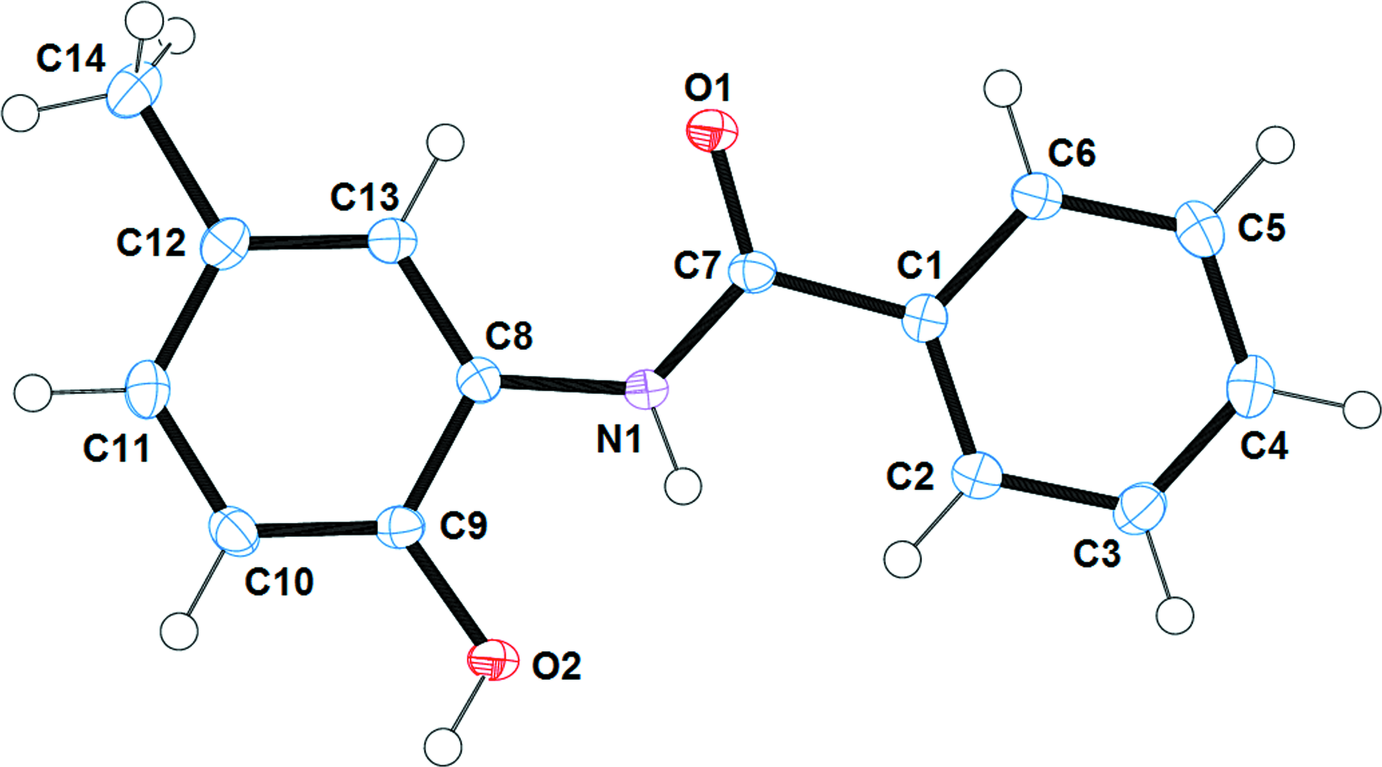

Supplement: Supplementary file 4 [file e-71-0o943-fig1.tif]

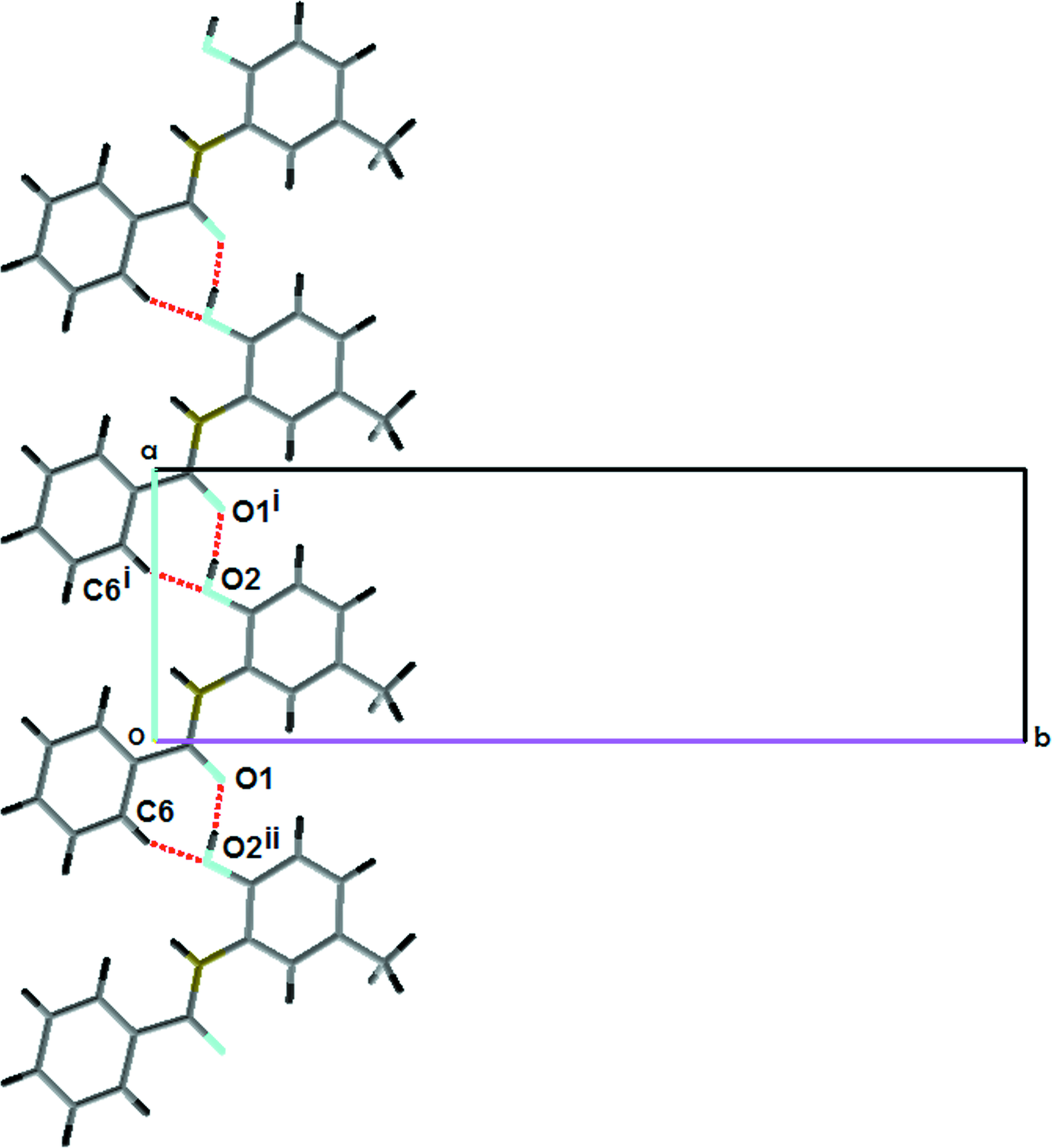

Supplement: Supplementary file 5 [file e-71-0o943-fig2.tif]

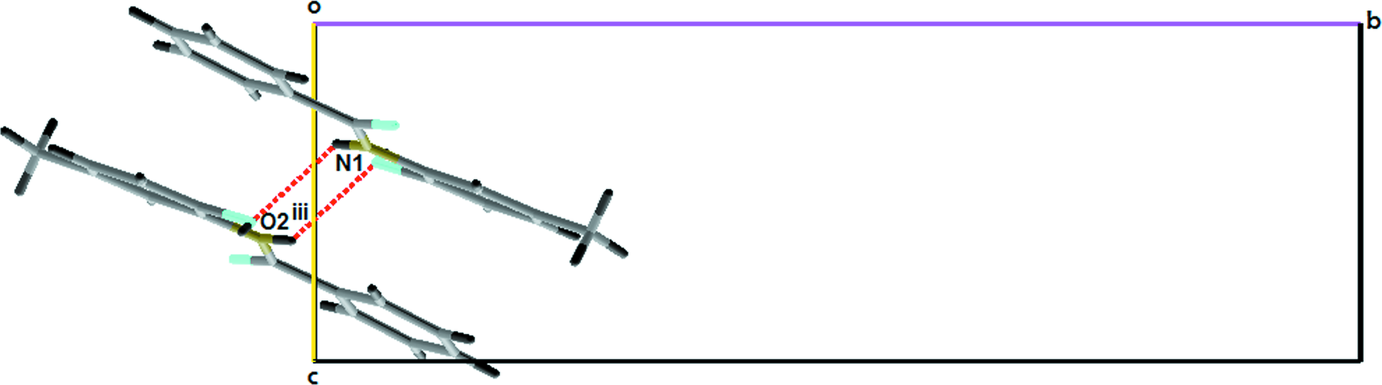

Supplement: Supplementary file 6 [file e-71-0o943-fig3.tif]
